# Evidence of malarial chemoprophylaxis among travellers who died from malaria: a systematic review and meta-analysis

**DOI:** 10.1186/s12936-023-04794-x

**Published:** 2023-11-25

**Authors:** Manas Kotepui, Kwuntida Uthaisar Kotepui, Frederick Ramirez Masangkay, Polrat Wilairatana

**Affiliations:** 1 Medical Technology, School of Allied Health Sciences, Walailak University, Tha Sala, Nakhon Si Thammarat, Thailand; 2 Department of Medical Technology, University of Santo Tomas, Manila, Philippines; 3 Department of Clinical Tropical Medicine, Faculty of Tropical Medicine, Mahidol University, Bangkok, Thailand

**Keywords:** Malaria, Chemoprophylaxis, Deaths, Compliance, Travelers

## Abstract

**Background:**

Chemoprophylaxis is a prevention method for malaria during travel in malaria-endemic countries. This study aimed to collate and synthesize the evidence of malarial chemoprophylaxis among malaria death cases.

**Methods:**

Studies documenting malarial chemoprophylaxis related to malaria deaths were searched in PubMed, Scopus, MEDLINE, Embase, and CENTRAL until 3 July 2022. The pooled proportion of malarial chemoprophylaxis among death cases was synthesized using logit transformation and back transformation to a proportion performed using generalized linear mixed models. The pooled log odds ratio (log-OR) with a 95% confidence interval (CI) of malarial chemoprophylaxis in death cases compared to survivors were synthesized.

**Results:**

Fifty-eight studies were included in the systematic review and the meta-analysis. Of 602 pooled malaria death cases, the number of patients who took chemoprophylaxis was 187 (30%) (95% CI 22–40, *P* < 0.01, 58 studies), and those who took adequate chemoprophylaxis were 24 (5%) (95% CI 2–13, *P* < 0.01, 42 studies). A comparable log-OR of underwent chemoprophylaxis was observed between malaria death cases and survivors (*P* = 0.94, pooled log-OR: − 0.02, 95% CI − 0.46–0.42, I^2^: 0%, 17 studies). Similarly, a comparable log-OR of adequate chemoprophylaxis was identified between malaria death cases and survivors (*P* = 0.15, pooled log-OR: 0.83, 95% CI − 0.30–1.97, I^2^: 47.08%, 11 studies).

**Conclusions:**

Among the studies where malarial chemoprophylaxis was reported, approximately 30% of malaria death cases had taken such prophylaxis. Notably, only 5% of these cases adhered fully or adequately to the recommended chemoprophylactic regimen. However, the analysis did not reveal a significant difference in the odds of malarial chemoprophylaxis between malaria death cases and survivors.

**Supplementary Information:**

The online version contains supplementary material available at 10.1186/s12936-023-04794-x.

## Background

Malaria is mainly caused by *Plasmodium falciparum*, and a lesser number is generated by *Plasmodium vivax*, *Plasmodium malariae*, *Plasmodium ovale*, and *Plasmodium knowlesi* through the bite of *Anopheles* mosquitoes [1–3]. In a non-endemic country, the Netherlands, approximately 94% of all *P. falciparum* infections were imported from Africa and were seen among travellers visiting friends and relatives (VFR) or asylum seekers [4]. Centers for Disease Control and Prevention (CDC) reported 2112 and 1788 imported malaria cases in the United States in 2017 and 2018, respectively [5, 6]. According to the systematic review and meta-analysis by Kendjo et al. [7]. France reported the highest number of imported malaria cases in Europe. The high number of imported malaria cases was attributed to increased international travellers and immigrants visiting malaria-endemic areas [7]. These populations are advised to take preventive measures for malaria, including anti-malarial chemoprophylaxis before, during, and after travelling to malaria-endemic areas [8–10]. Nevertheless, the recent meta-analysis showed a high proportion of severe malaria cases even with good adherence to the malarial chemoprophylaxis [11]. However, patients who had taken chemoprophylaxis appeared to have a lower proportion of severe malaria than those who did not [12–15]. In countries with known chloroquine-resistant *P. falciparum* malaria, inappropriate malaria chemoprophylaxis, such as taking chloroquine alone or with proguanil for malaria chemoprophylaxis, led to malaria deaths [16]. In the UK, malaria mortality increased steadily with age, with no deaths in children under 5 years; meanwhile, 4.6% of deaths were reported in people over 65 years [17]. A higher case fatality was observed among tourists (3%) compared to VFR (0.32%) [17].

Compliance with malaria chemoprophylaxis is the most important strategy for preventing malaria in travellers. However, the inappropriate use or early discontinuation of chemoprophylaxis can increase the risk of contracting malaria, which may lead to severe malaria and even death in the case of delayed diagnosis and treatment. In this study, the evidence of anti-malarial chemoprophylaxis among malaria death cases was retrospectively collated and synthesized. In addition, the compliance of anti-malaria chemoprophylaxis between malaria death cases and survivors was retrospectively collated and synthesized using the meta-analysis approach. The findings of this study can support a better understanding of the anti-malarial chemoprophylaxis use and its correlation to the risk of death among travellers to malaria-endemic areas.

## Methods

This systematic review and meta-analysis were conducted according to Preferred Reporting Items for Systematic Reviews and Meta-analyses (PRISMA) recommendations (PRISMA Abstract Checklist, PRISMA 2020 Checklist). The systematic review was registered at PROSPERO with registration number: CRD42022352353.

### Data sources and searches

Studies documenting chemoprophylaxis related to malaria mortality were searched in PubMed, Scopus, MEDLINE, Embase, and CENTRAL until 3 July 2022. The search strategy included the keywords: chemoprophylaxis OR chemoprevention OR antimalaria* OR anti-malaria* OR “anti malarial” OR “anti malaria” OR prophylaxis OR prophylactic OR “malaria prevention” OR “malarial prevention”) AND (malaria OR Plasmodium OR “remittent fever” OR “marsh fever” OR paludism) AND (traveler OR travel OR imported OR immigrant* OR emigrant* OR foreigner) AND (died OR dead OR mortality OR fatality OR death). The searches have broadened by incorporating additional databases, such as Google Scholar and previous reviews on imported malaria, to include additional relevant studies that were not identified by the database search. The complete description of the literature search strategies and filters is available in Additional file 1: Table S1.

### Definitions of malarial chemoprophylaxis

Malarial chemoprophylaxis was judged to be correct when administering medications according to World Health Organization guideline [18]. Complete or adequate use was recorded as self-reported and defined as regular, continuous prophylactic medications as recommended before travel and up to the interview of the study [19].

### Eligibility criteria

The PICO (P: population, I: intervention, C: comparators, O: outcome) method was used to identify studies that met the inclusion criteria. P: data on malaria deaths and chemoprophylaxis. I: none; C: those with chemoprophylaxis data who survived malaria; O: the prevalence or proportion of malaria deaths involving those who used chemoprophylaxis. All studies published in English that reported chemoprophylaxis use among malaria death cases, including case reports and case series, were included. Moreover, the following^s^ studies were excluded: (i) studies that did not report chemoprophylaxis data for malaria mortality, (ii) research that reported on chemoprevention but did not include cases of malaria deaths, (iii) malaria cases without history of travelling or occurring among migrant population, (iv) review articles, (v) conference abstracts, and (vi) studies without a comparison group.

### Study selection and data extraction

EndNote (version 20, Stanford, CT, USA) was used to manage studies retrieved from databases and other sources. After removing duplicate research automatically and manually, two independent authors (MK, KUK) reviewed the titles and abstracts of the remaining studies. Second, non-relevant titles and abstracts were removed. Thirdly, the full text of possibly relevant studies was reviewed, and ineligible papers were excluded for specific reasons. The following data were extracted from eligible studies using a pre-prepared Excel spreadsheet: Author(s) and year of publication, year of study, country, study design, number of participants, *Plasmodium* spp., age, sex proportion, number of malaria death cases, number of deaths (who took chemoprophylaxis, adequate chemoprophylaxis, inadequate chemoprophylaxis, and no chemoprophylaxis), number of survivors, the proportion of survivors (who took chemoprophylaxis, adequate chemoprophylaxis, inadequate chemoprophylaxis, no chemoprophylaxis), the visiting country, and diagnostic test for malaria. Two authors (MK and KUK) independently extracted the data. And any differences or conflicts between the two authors were resolved through consensus.

### Quality assessment

The review included studies with various designs, such as prevalence (cross-sectional) studies, case-control studies, cohort studies, case reports, and case series. Due to the diverse nature of these designs, a direct comparison of their quality was not feasible. Consequently, no quality assessment was performed in this review.

### Data analysis

To synthesize the estimated pooled proportion, the number of malaria death cases who took chemoprophylaxis (n), and the total number of malaria death cases with chemoprophylaxis data (N) were used. Logit transformation and back transformation to a proportion were performed using generalized linear mixed models (GLMMs). The meta-analyses of proportion studies were conducted using the command “metaprop_one” in the Stata version 17.0 software (Stata Corp., College Station, Texas, USA) as described previously [20], dealing with zero cases of chemoprophylaxis among malaria deaths. To synthesize the pooled log odds ratio (OR) with a 95% confidence interval (CI), the number of malaria death cases who took chemoprophylaxis, the total number of malaria death cases whose chemoprophylaxis data were available, the number of survivors who took chemoprophylaxis, and a total number of survivors whose chemoprophylaxis data were available, were used. The pooled proportion estimate and 95% CI were calculated using the DerSimonian-Laird method with the random-effects model based on the inverse variance method for measuring the weight [21]. Chi-square (Q) test was used to determine the significant heterogeneity across the studies, with *P* < 0.05 indicating significant heterogeneity [22, 23]. Publication bias was assessed by visual inspection of the funnel plot, Egger’s regression test, and Contour-enhanced funnel plot as described previously [22, 23]. A univariate regression analysis was performed based on publication year, study area, study design, and age of patients as covariates. Moreover, a subgroup analysis was conducted based on the covariate that significantly confounded the pooled estimate by the meta-regression analysis. To determine whether the total pooled estimate was unaffected by a single study, a sensitivity analysis was conducted by excluding a single study and reran the meta-analysis.

## Results

### Search results

**Fig. 1:**
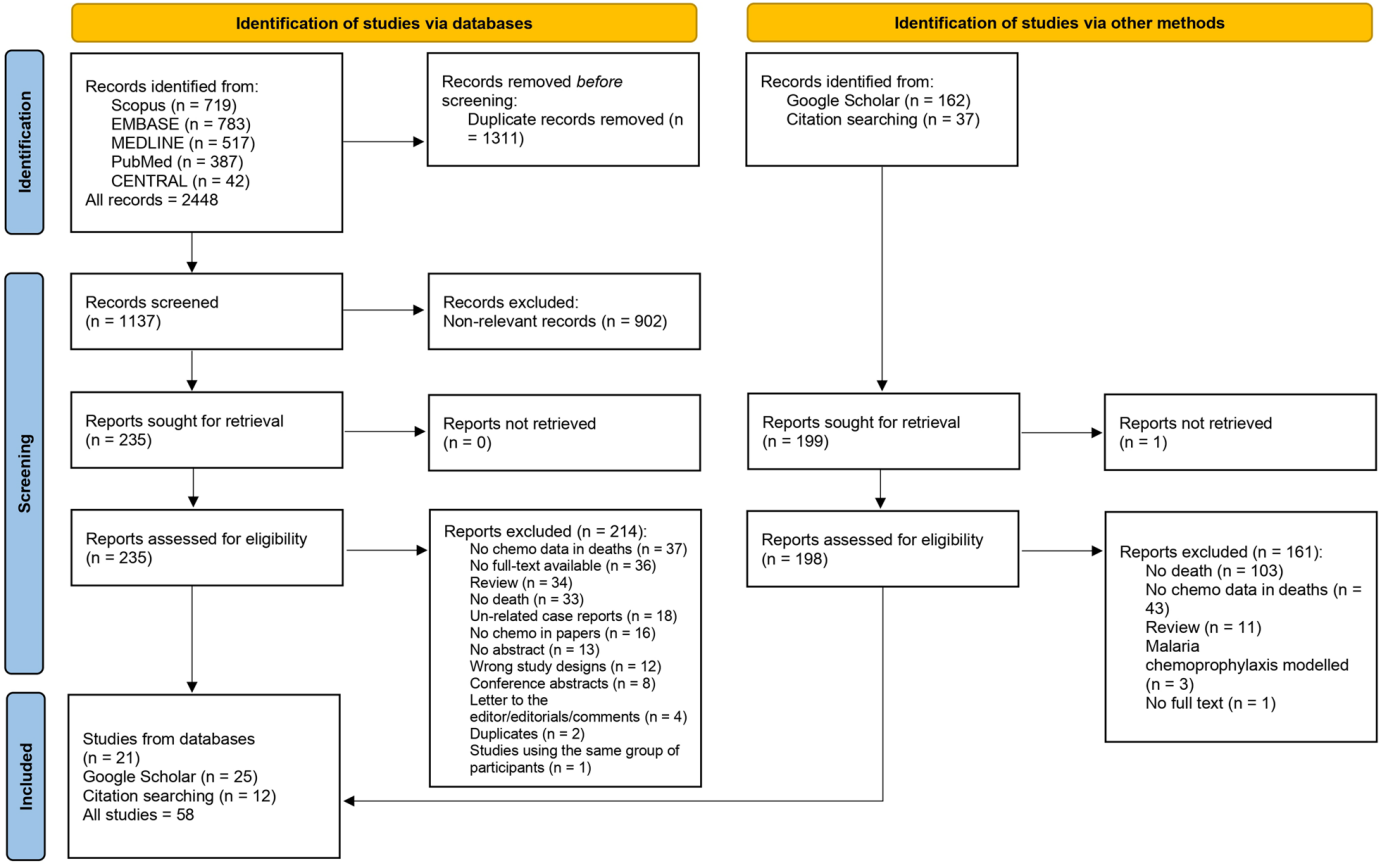
Study flow diagram

A total of 2448 studies were retrieved from five databases: Scopus (n = 719), EMBASE (n = 783), MEDLINE (n = 517), PubMed (n = 387), and CENTRAL (n = 42). After removing 1311 duplicated articles, 1137 were screened for titles and abstracts. A total of 902 non-relevant studies was excluded, and the remaining 235 studies were examined for full text. Then, 214 studies were excluded for specific reasons, and 21 studies [17, 24–43] that met the eligibility criteria were included in the review. Of 162 studies identified from Google Scholar, 25 [4, 12, 44–66] that met the eligibility criteria were also included. Twelve studies [5, 67–77] were identified from reference lists of the included studies and review articles. Finally, 58 studies [4, 5, 12, 16, 17, 25–77] were included in the systematic review (Fig. 1).

### Characteristics of the included studies

Characteristics of the included studies are summarized in Table 1. Most studies were published between 2000 and 2009 (26 studies, 44.8%), conducted in North America (30 studies, 51.7%), cross-sectional studies (34 studies, 58.6%), patients with *P. falciparum* and other *Plasmodium* species (43 studies, 74.1%), patients in all age groups (34 studies, 58.6%), using microscopy for malaria diagnosis (28 studies, 48.283%). Details of the included studies are shown in Additional file 2: Table S2.

**Table 1:** Characteristics of the included studies

Characteristics	Number	Percent
Publication years		
Before 2000	9	15.52
2000–2009	26	44.83
2010–2022	23	39.66
Continents		
Asia	1	1.72
Europe	27	46.5
North America	30	51.72
Study designs		
Case reports/Case series	4	6.89
Cohort studies	2	3.45
Cross-sectional studies	34	58.62
Prospective observational studies	4	6.90
Retrospective observational studies	14	24.14
*Plasmodium* species		
*P. falciparum* only	14	24.04
*P. vivax* only	1	1.7
*P. falciparum* and other species	43	74.14
Age groups		
Children	1	1.72
Adults	15	25.26
Children and adults	1	1.72
All age groups	34	58.62
Not specified	7	12.07
Diagnostic methods for malaria		
Microscopy	28	48.28
Microscopy or PCR	2	3.4
Microscopy or RDT	6	10.34
Microscopy or RDT or PCR	8	13.79
Hospital physicians	1	1.72
Not specified	13	22.41

*PCR* polymerase chain reaction, *RDT* rapid diagnostic test

### Global distribution of malaria deaths and countries visited by infected individuals

**Fig. 2:**
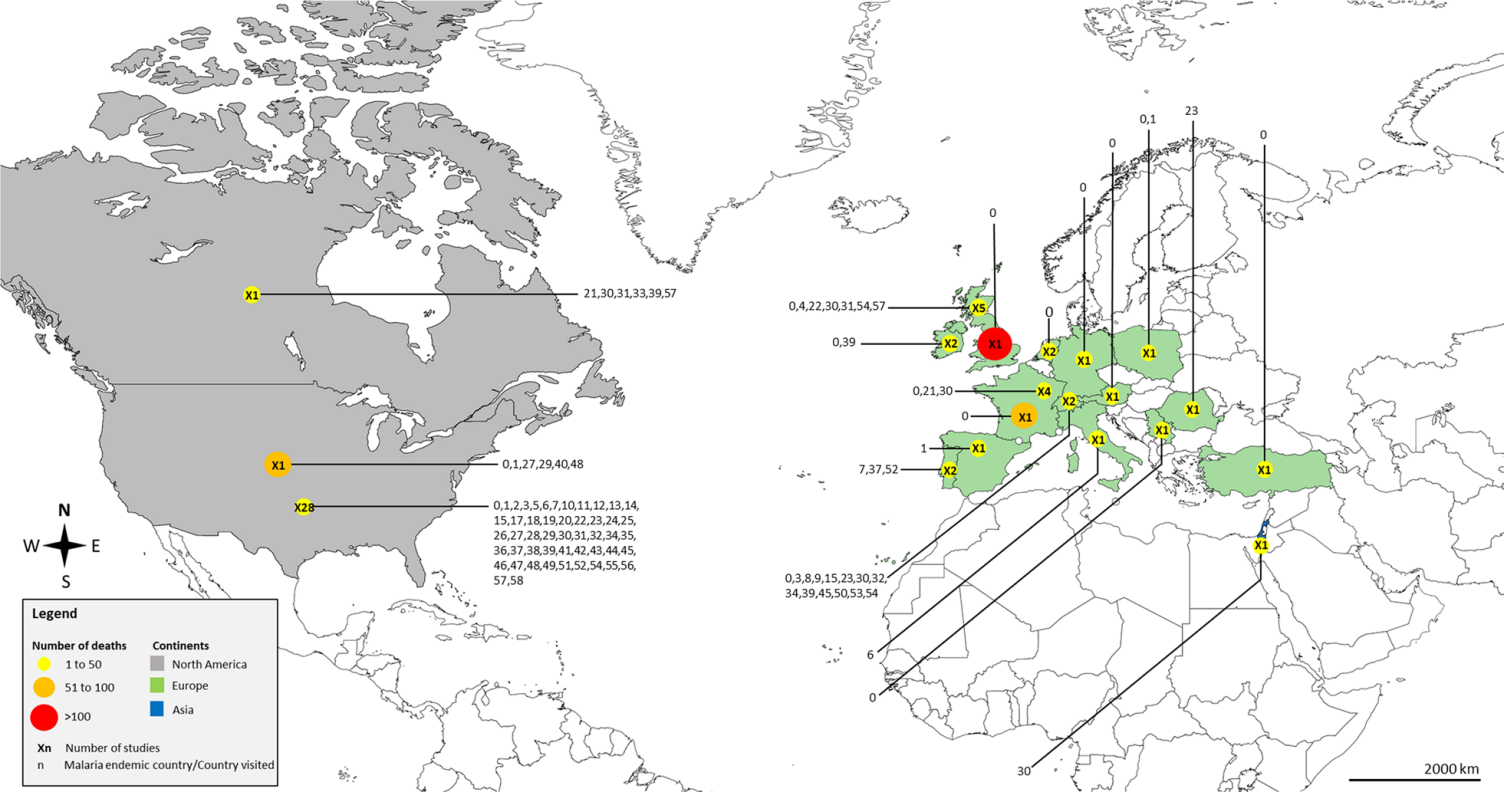
Global distribution of imported malaria deaths and countries visited by infected individuals. **1** Africa; **2** Africa (East); **3** Africa (South); **4** Africa (West); **5** Africa (Central); **6** Sub-Saharan Africa; **7** Angola; **8** Benin; **9** Botswana; **10** Burkina Faso; **11** Cameroon; **12** Cape Verde; **13** Chad; **14** China; **15** Democratic Republic of Congo (Zaire); **16** Dominican Republic; **17** Ecuador; **18** Egypt; **19** Equatorial Guinea; **20** Ethiopia; **21** Gabon; **22** Gambia; **23** Ghana; **24** Guatemala; **25** Guinea; **26** Guyana; **27** Haiti; **28** Honduras; **29** India; **30** Kenya; **31** Liberia; **32** Madagascar; **33** Malawi; **34** Mali; **35** Mauritius; **36** Mexico; **37** Mozambique; **38** Nicaragua; **39** Nigeria; **40** Papua New Guinea; **41** Philippines; **42** Puerto Rico; **43** Republic of Congo; **44** Rwanda; **45** Senegal; **46** Sierra Leone; **47** Somalia; **48** South America; **49** Sudan; **50** Switzerland; **51** Tanzania; **52** Thailand; **53** Togo; **54** Uganda; **55** United States of America (USA); **56** Yemen; **57** Zambia; **58** Zimbabwe. The map was generated by authors using the map freely available at https://mapchart.net/. Authors are allowed to use, edit and modify any map created with mapchart.net for publication freely by adding the reference to mapchart.net

Global distribution of malaria deaths and countries visited by infected individuals was demonstrated using data on malaria death reported by the included studies (Additional file 2: Table S2). Most malaria deaths were reported among individuals that visited African countries and returned to North America and European countries (Fig. 2).

### The proportion of malaria death cases who took any chemoprophylaxis

**Fig. 3:**
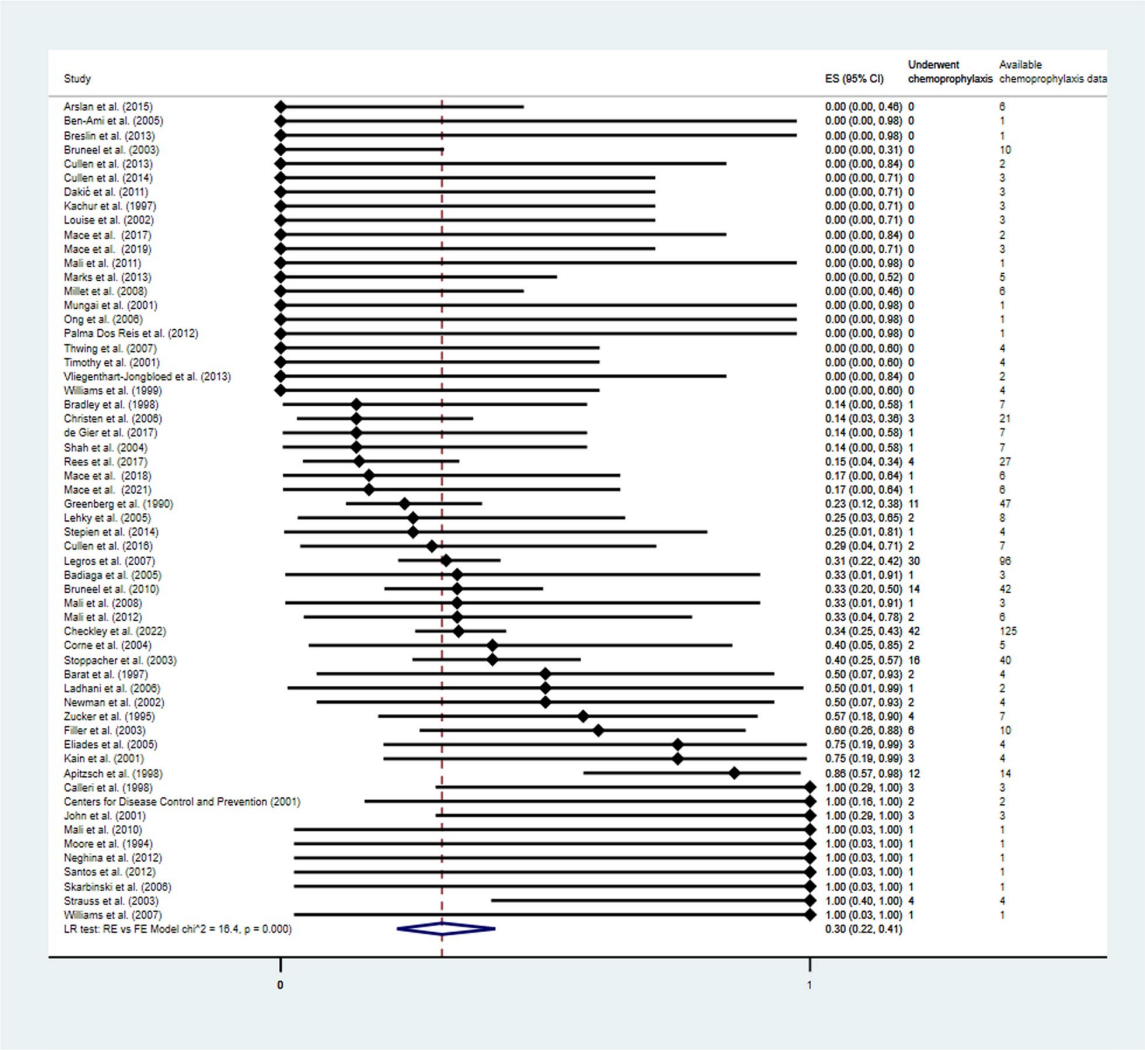
The pooled proportion of malaria death cases who took any chemoprophylaxis

According to the findings of the 58 studies [4, 5, 12, 16, 17, 25–77] with 602 malaria death cases, and 187 took chemoprophylaxis. Meta-analysis results showed that the pooled proportion of malaria death cases who took any chemoprophylaxis was 30% (95% CI 22–40, *P* < 0.01, 58 studies, Fig. 3). The subgroup analysis by years of publication showed that the pooled proportion of malaria death cases who took any chemoprophylaxis before 2000 was 43% (95% CI 17–73, *P* < 0.01, 9 studies, Additional file 1: Fig. S1), during 2000–2009 was 32% (95% CI 20–49, *P* < 0.01, 26 studies), and during 2010–2022 was 22% (95% CI 14–33, *P* = 0.08, 23 studies).

### The proportion of malaria death cases who took complete or adequate chemoprophylaxis

**Fig. 4:**
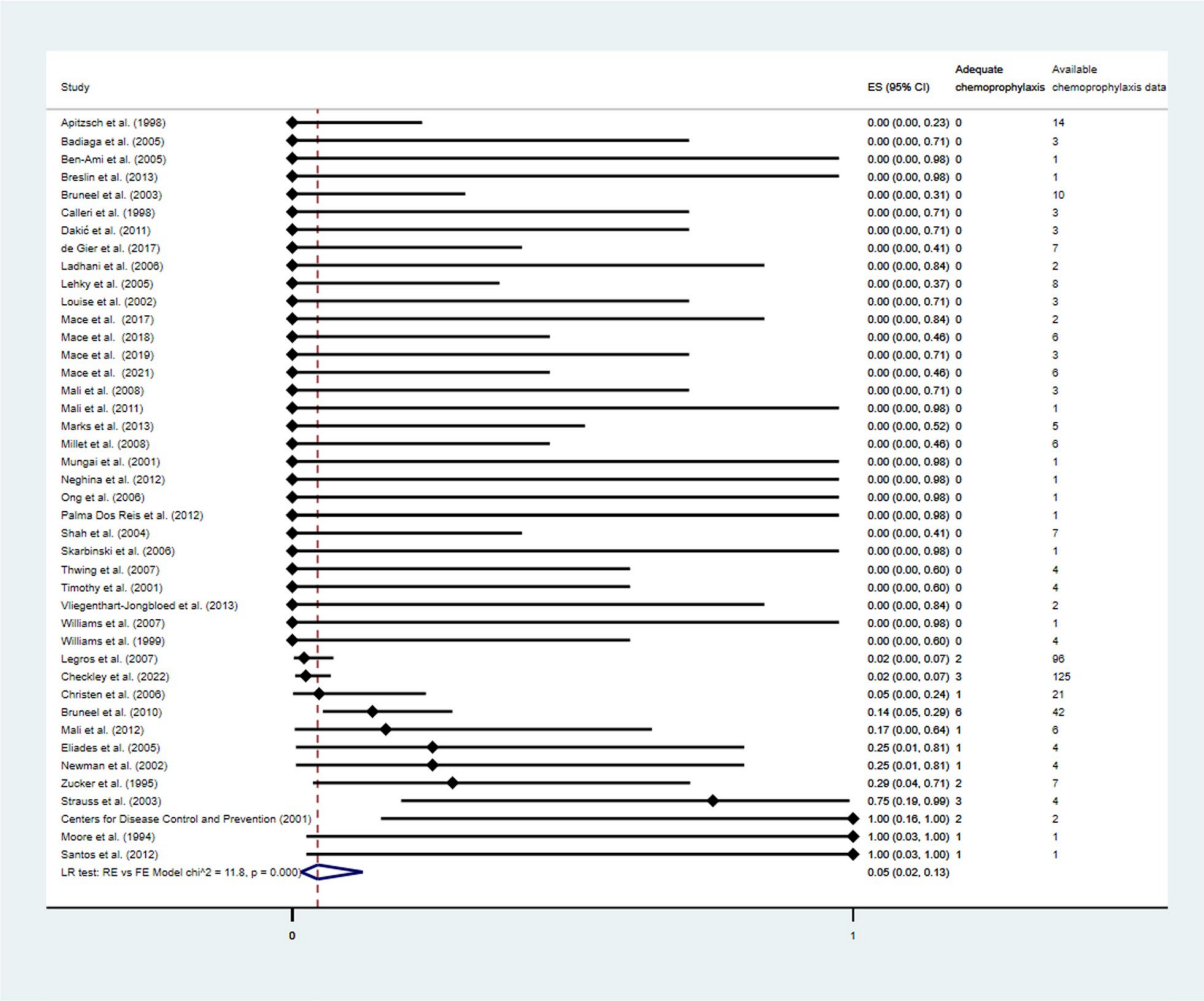
The pooled proportion of malaria death cases who took adequate chemoprophylaxis

Among 58 studies included in the systematic review, 42 [4, 5, 12, 16, 17, 26, 28–31, 33, 35–42, 44, 46, 50–53, 56–60, 62, 64–67, 71–77] reported malaria deaths were 602 and of which included 24 cases who took adequate chemoprophylaxis. Therefore, the pooled proportion of malaria death cases who took adequate chemoprophylaxis was 5% (95% CI 2–13, *P* < 0.01, 42 studies, Fig. 4). The subgroup analysis by years of publication showed that the pooled proportion of malaria death cases who took adequate chemoprophylaxis before 2000 was 4% (95% CI 0–89, *P* = 0.10, 5 studies, Additional file 1: Fig. S2), during 2000–2009 was 3% (95% CI 0–18, *P* < 0.01, 21 studies), and during 2010–2022 was 5% (95% CI 2–13, *P* = 0.05, 16 studies).

### The proportion of malaria death cases who took no chemoprophylaxis

Of 602 pooled malaria death cases, 344 did not take any chemoprophylaxis, according to the 58 studies [4, 5, 12, 16, 17, 25–77]. Meta-analysis results showed that the pooled proportion of malaria death cases who did not take any chemoprophylaxis was 67% (95% CI 53–79, *P* < 0.01, 58 studies, Additional file 1: Fig. S3). The subgroup analysis by years of publication showed that the pooled proportion of malaria death cases who did not take any chemoprophylaxis before 2000 was 60% (95% CI 42–76, *P* = 0.08, 9 studies, Additional file 1: Fig. S4) during 2000–2009 was (63%, 95% CI 38–83, *P* < 0.01, 26 studies), and during 2010–2022 was 78% (95% CI 67–86, *P* = 0.03, 23 studies).

### Odds of chemoprophylaxis in malaria death cases and survivors

The pooled log-OR of chemoprophylaxis in malaria death cases and survivors was estimated using the data of 17 studies [12, 25, 26, 28–33, 37, 39, 41, 53, 54, 58, 71, 75]. The meta-analysis results showed a comparable log-OR of chemoprophylaxis between malaria death cases and survivors (*P* = 0.94, pooled log-OR: − 0.02, 95% CI − 0.46–0.42, I^2^: 0%, 17 studies, Additional file 1: Fig. S5). Meta-regression analysis showed that publication year (*P* = 0.55), continents (*P* = 0.93), and study design (*P* = 0.92) did not confound the pooled effect estimate. Therefore, subgroup analyses of these covariates were not further performed.

### Odds of complete or adequate chemoprophylaxis in deaths and survivors

The pooled log-OR of adequate chemoprophylaxis in malaria death cases and survivors was estimated using the data from 11 studies [12, 26, 28–31, 37–39, 53, 75]. The meta-analysis results showed a comparable log-OR of adequate chemoprophylaxis between malaria death cases and survivors (*P* = 0.21, pooled log-OR: 0.60, 95% CI − 0.35–1.55, I^2^: 26.81%, 11 studies, Additional file 1: Fig. S6). The meta-regression analysis showed that publication year (*P* = 0.60), continents (*P* = 0.40), and study design (*P* = 0.55) did not confound the pooled effect. Therefore, subgroup analyses of these covariates were not further performed.

### Sensitivity analysis

The leave-one-out method was used to identify outliers in the meta-analysis. After each study was excluded and reran the meta-analysis of the pooled log-OR, a comparable log-OR of undergoing chemoprophylaxis was observed between malaria death cases and survivors (*P* > 0.05 in each reran the analysis, Additional file 1: Fig. S7). Moreover, a comparable log-OR of adequate chemoprophylaxis was identified between malaria death cases and survivors (*P* > 0.05 in each reran the analysis, Additional file 1: Fig. S8). These results indicated that the meta-analysis results were robust.

### Publication bias

For the meta-analysis of odds of chemoprophylaxis in malaria death cases and survivors, the funnel plot was symmetrical (Additional file 1: Fig. S9); Egger’s test showed no significant difference in small-study effect (*P* = 0.92). For the meta-analysis of odds of adequate chemoprophylaxis in malaria death cases and survivors, the funnel plot was symmetrical (Additional file 1: Fig. S10); Egger’s test showed no significant difference in small-study effect (*P* = 0.17). These results suggested no publication bias of the pooled effect estimates.

## Discussion

The main finding of this study was that 30% of malaria deaths among travellers occurred in those who used malarial chemoprophylaxis. Low/Poor compliance with malarial chemoprophylaxis was observed among malaria deaths. Malaria deaths among travellers have been linked to noncompliance with malarial chemoprophylaxis and treatment delay [67]. The effect of chemoprophylaxis in malaria mortality cases is uncertain due to the low number of case fatalities reported in the literature. This systematic review and meta-analysis indicated that 30% of malaria death cases took malarial chemoprophylaxis. However, a low proportion of malaria deaths (5%) took complete or adequate malarial chemoprophylaxis suggesting that noncompliance might increase the risk of getting malaria, the risk of severe disease, and subsequently, deaths among travellers and immigrants. Vliegenthart-Jongbloed et al. [4] showed that travellers of African ethnicity predominated the group who did not use the chemoprophylaxis. The low adherence to malarial chemoprophylaxis among African VFRs was due to the financial problem of purchasing malaria chemoprophylaxis [78, 79] or complex cultural factors [80].

Although chemoprophylaxis compliance is associated with reduced malaria severity as demonstrated by previous studies [4, 81], no difference in malarial chemoprophylaxis between malaria death cases and survivors was observed. Vliegenthart-Jongbloed et al. [4] demonstrated that patients who adhere to chemoprophylaxis acquire non-falciparum malaria more frequently, have significantly lower *P. falciparum* parasitaemia on admission, and have lower odds of severe malaria than patients who do not adhere to chemoprophylaxis. This suggested that malarial chemoprophylaxis provides protection against *P. falciparum* infection and severe disease. For non-*falciparum* malaria, such as *P. vivax* infection, chemoprophylaxis is effective against blood-stage parasites but ineffective against late, hypnozoite reactivation-related attacks, and patients might have a delayed onset of illness [82, 83].

Several factors contributed to malaria deaths among travellers and immigrants. Increasing age was an independent risk factor for severe malaria and death due to the decreasing immune response against *Plasmodium* infections [84, 85]. Pregnancy is another risk factor for malaria deaths because of the reduced immune responses [86]. Moreover, early diagnosis and appropriate treatment significantly reduce malaria-related mortality [87, 88]. Therefore, the delayed presentation may be the most critical factor leading to high mortality and providing the required information to the traveller on how to respond in the event of illness upon return. Another risk of malaria deaths is the missed malaria diagnosis during the first presentation. Malaria signs and symptoms are frequently misdiagnosed as influenza-like viral syndromes. Lastly, changes in the population structure are another risk factor for mortality. Previous studies showed that Africans with severe malaria had a lower risk of death than Europeans with severe malaria due to partial immune protection [17, 36]. It is possible that there were fewer reports about taking anti-malarial prophylaxis among VFRs compared with other travellers as they might not be drug accessible before travel or lacked knowledge and perception about the risk of getting malaria [43]. In some countries, no prophylaxis is recommended according to the national guideline. Migrants/immigrants and travellers may show more severe infections than residents in endemic areas. It may depend on the malaria endemicity of the original place and travel/re-settlement areas. Nevertheless, the clinical presentation of malaria may be milder in migrants than in travellers due to the semi-immunity to malaria among immigrants [89], which contributes to a lower risk of death.

In light of the results of this study, which revealed that only 5% of individuals achieved full or adequate adherence to malarial chemoprophylaxis—falling below the established threshold of above 90% adherence for effective malaria chemoprophylaxis in travel medicine—it becomes paramount to prioritize education and awareness efforts. These efforts should be targeted at travellers and immigrants visiting malaria-endemic regions, highlighting the critical importance of rigorous adherence to prescribed prophylactic regimens. Moreover, it is crucial to recognize and address the challenges associated with achieving high adherence rates, including financial constraints and cultural factors. Looking ahead, future research in travel medicine should not only investigate the determinants of adherence but also explore effective interventions aimed at promoting and monitoring adherence to malarial chemoprophylaxis. Such endeavours will undoubtedly contribute to the enhancement of malaria prevention strategies within these vulnerable populations.

While the findings of this study highlight the potential risks associated with noncompliance, it is essential to acknowledge the study’s limitations. The inclusion criteria focused on studies that reported on the use of chemoprophylaxis, which may introduce bias and limit the generalizability of findings. Therefore, the conclusion that could be drawn is specific to those studies where prophylaxis was reported, and it is not representative of the entire population of malaria death cases. Furthermore, the analysis encompassed a mix of studies, including clinical cases, which introduced heterogeneity into our findings. Another limitation is the small number of articles that consider prescription or adherence to chemoprophylaxis, which implies little representativeness and may compromise the validity of the results. Furthermore, it is essential to consider the evaluation of malaria chemoprophylaxis, including its prescription, appropriateness, and adherence, as a critical aspect of this study. While it is relatively straightforward to assess whether prophylaxis was prescribed or if inappropriate medication was prescribed, evaluating adherence to prophylaxis presents a significant challenge. The limitation of this study lies in the difficulty of accurately evaluating adherence among travellers and immigrants. This limitation is particularly pertinent when trying to establish a clear association between malaria chemoprophylaxis and malaria death cases. The complexity of assessing adherence to malaria chemoprophylaxis can lead to the finding that there was no statistically significant difference in the use of chemoprophylaxis between malaria death cases and survivors. It is plausible that noncompliance with prescribed prophylaxis played a role in some malaria death cases, but due to the limitations in data availability, this study was unable to comprehensively evaluate this aspect.

The present analysis relied on existing literature, which often did not provide comprehensive information on crucial prophylaxis-related factors, such as the source of prophylaxis, the specific drug used, dosage, and the duration of prophylaxis. These data gaps limit the ability to perform a more detailed evaluation of the relationship between chemoprophylaxis and malaria mortality. Furthermore, the present study did not fully account for potential confounders that could influence malaria-related mortality, such as the age of individuals or delays in diagnosis. These confounding factors may play a significant role in the observed outcomes but were not incorporated into the analysis. Lastly, while this study aimed to provide a comprehensive overview of adherence to malarial chemoprophylaxis based on the available literature, the absence of such detailed data is indeed a limitation. It is important to recognize that different anti-malarial drugs may have varying levels of efficacy and adherence, which could influence the outcomes observed. Future investigations should aim to collect detailed data on prophylaxis-related factors, potential confounders, and the specific drugs used for chemoprophylaxis to provide a more comprehensive understanding of the complex dynamics influencing malaria mortality among travellers and immigrants. Such research endeavours will contribute to the refinement of malaria prevention strategies and ultimately enhance the well-being of these vulnerable populations.

## Conclusion

Among the studies where malarial chemoprophylaxis was reported, approximately 30% of malaria death cases had taken such prophylaxis. Notably, only 5% of these cases adhered fully or adequately to the recommended chemoprophylactic regimen. No difference in the odds of anti-malarial chemoprophylaxis between malaria death cases and survivors was observed. Therefore, besides chemoprophylaxis, other factors may contribute to malaria deaths. As most of malaria deaths did not involve appropriate malarial chemoprophylaxis, educating travellers and immigrants who travel to malaria-endemic areas about malaria prevention and chemoprophylaxis is crucial to reduce malaria mortality.

### Supplementary Information

**Additional file 1.** Table S1, Fig. S1–S10.

**Additional file 2.** Table S2.

## Data Availability

All data relating to the present study are available in this manuscript and Additional files.
