# Supplementary material for: Evidence of malarial chemoprophylaxis among travellers who died from malaria: a systematic review and meta-analysis
Source: Malar J. 2023 Nov 25;22:359. doi: 10.1186/s12936-023-04794-x (PMC10675877; doi:10.1186/s12936-023-04794-x)
Supplement: Supplementary file 1 — Additional file 1. Table S1, Fig. S1–S10. [file 12936_2023_4794_MOESM1_ESM.pdf]

Evidence of malarial chemoprophylaxis adherence among travelers who died from malaria: A systematic review and meta-analysis

Manas Kotepui[1*], Kwuntida Uthaisar Kotepui[1], Frederick Ramirez Masangkay[2], Polrat Wilairatana[3*]

[1] Medical Technology, School of Allied Health Sciences, Walailak University, Tha Sala, Nakhon Si Thammarat, Thailand

[2] Department of Medical Technology, University of Santo Tomas, Manila, Philippines;

[3] Department of Clinical Tropical Medicine, Faculty of Tropical Medicine, Mahidol University, Bangkok, Thailand

[4]Department of Global Health, National Centre for Epidemiology and Population Health, College of Health & Medicine, Australian National University, Canberra, Acton, ACT 2601, Australia

[*]**Corresponding author**

Manas Kotepui manas.ko@wu.ac.th, Tel+ :.66954392469

Kwuntida Uthaisar Kotepui: kwuntida.ut@wu.ac.th

Frederick Ramirez Masangkay: frederick_masangkay2002@yahoo.com

Polrat Wilairatana: polrat.wil@mahidol.ac.th

Additional file 1

Table S1. Search strategy

Scopus

3 July 2022

No.	Query	Results
#5	#1 AND #2 AND #3 AND #4 AND #5	719
#4	TITLE-ABS-KEY (chemoprophylaxis OR chemoprevention OR antimalaria* OR anti-malaria* OR "anti malarial" OR "anti malaria" OR prophylaxis OR prophylactic OR "malaria prevention" OR "malarial prevention")	374407
#3	TITLE-ABS-KEY (malaria OR plasmodium OR "remittent fever" OR "marsh fever" OR paludism)	149841
#2	TITLE-ABS-KEY (died OR dead OR mortality OR fatality OR death)	3148745
#1	TITLE-ABS-KEY (traveler OR travel OR imported OR immigrant* OR emigrant* OR foreigner)	445155

EMBASE

3 July 2022

No.	Query	Results
#5	#1 AND #2 AND #3 AND #4	783
#4	died OR 'dead'/exp OR dead OR 'mortality'/exp OR mortality OR 'fatality'/exp OR fatality OR 'death'/exp OR death	3330094
#3	'traveler'/exp OR traveler OR 'travel'/exp OR travel OR imported OR immigrant* OR emigrant* OR 'foreigner'/exp OR foreigner	164149
#2	'malaria'/exp OR malaria OR 'plasmodium'/exp OR plasmodium OR 'remittent fever' OR 'marsh fever'/exp OR 'marsh fever' OR 'paludism'/exp OR paludism	156722
#1	'chemoprophylaxis'/exp OR chemoprophylaxis OR 'chemoprevention'/exp OR chemoprevention OR antimalaria* OR 'anti malaria*' OR 'anti malarial' OR 'anti malaria' OR 'prophylaxis'/exp OR prophylaxis OR prophylactic OR 'malaria prevention'/exp OR 'malaria prevention' OR 'malarial prevention'	1338473

MEDLINE

Databases	Search terms/Search strategy	Date
MEDLINE	(chemoprophylaxis OR chemoprevention OR antimalaria* OR anti-malaria* OR “anti malarial” OR “anti malaria” OR prophylaxis OR prophylactic OR “malaria prevention” OR “malarial prevention”)	3 July 2022

	AND (malaria OR Plasmodium OR “remittent fever” OR “marsh fever” OR paludism) AND (traveler OR travel OR imported OR immigrant* OR emigrant* OR foreigner) AND (died OR dead OR mortality OR fatality OR death)<br>Search results :517	

CENTRAL

3 July 2022

**ID**	**SearchHits**	**Results**
#5	#1 AND #2 AND #3 AND #4	42
#4	died OR dead OR mortality OR fatality OR death	162925
#3	traveler OR travel OR imported OR immigrant* OR emigrant* OR foreigner	6364
#2	malaria OR Plasmodium OR “remittent fever” OR “marsh fever” OR paludism	7420
#1	chemoprophylaxis OR chemoprevention OR antimalaria* OR anti-malaria* OR “anti malarial” OR “anti malaria” OR prophylaxis OR prophylactic OR “malaria prevention” OR “malarial prevention”	46204

PubMed

3 July 2022

Search number	Query	Search Details	Results
5	#1 AND #2 AND #3 AND #4	("chemoprevention"[MeSH Terms] OR "antimalarials"[MeSH Terms]) AND ("malaria"[MeSH Terms] OR "plasmodium"[MeSH Terms]) AND ("travel"[MeSH Terms] OR "travel"[All Fields] OR "traveling"[All Fields] OR "travelling"[All Fields] OR "travels"[All Fields] OR "traveled"[All Fields] OR "traveler"[All Fields] OR "traveler s"[All Fields] OR "travelers"[All Fields] OR "travelled"[All Fields] OR "traveller"[All Fields] OR "traveller s"[All Fields] OR "travellers"[All Fields] OR ("travel"[MeSH Terms] OR "travel"[All Fields] OR "traveling"[All Fields] OR "travelling"[All Fields] OR "travels"[All Fields] OR "traveled"[All Fields] OR "traveler"[All Fields] OR "traveler s"[All Fields] OR "travelers"[All Fields] OR "travelled"[All Fields] OR "traveller"[All Fields] OR "traveller s"[All Fields] OR "travellers"[All	387

		Fields]) OR ("import"[All Fields] OR "importation"[All Fields] OR "importations"[All Fields] OR "imported"[All Fields] OR "importer"[All Fields] OR "importers"[All Fields] OR "importing"[All Fields] OR "imports"[All Fields]) OR "immigrant*"[All Fields] OR "emigrant*"[All Fields] OR ("emigrants and immigrants"[MeSH Terms] OR ("emigrants"[All Fields] AND "immigrants"[All Fields]) OR "emigrants and immigrants"[All Fields] OR "foreigner"[All Fields] OR "foreigners"[All Fields])) AND ("death"[MeSH Terms] OR "death"[All Fields] OR "died"[All Fields] OR ("death"[MeSH Terms] OR "death"[All Fields] OR "dead"[All Fields]) OR ("mortality"[MeSH Terms] OR "mortality"[All Fields] OR "mortalities"[All Fields] OR "mortality"[MeSH Subheading]) OR ("fatal"[All Fields] OR "fatalities"[All Fields] OR "fatality"[All Fields] OR "fatally"[All Fields]) OR ("death"[MeSH Terms] OR "death"[All Fields] OR "deaths"[All Fields]))	
4	died OR dead OR mortality OR fatality OR death	"death"[MeSH Terms] OR "death"[All Fields] OR "died"[All Fields] OR "death"[MeSH Terms] OR "death"[All Fields] OR "dead"[All Fields] OR "mortality"[MeSH Terms] OR "mortality"[All Fields] OR "mortalities"[All Fields] OR "mortality"[MeSH Subheading] OR "fatal"[All Fields] OR "fatalities"[All Fields] OR "fatality"[All Fields] OR "fatally"[All Fields] OR "death"[MeSH Terms] OR "death"[All Fields] OR "deaths"[All Fields]	2,485,819
3	traveler OR travel OR imported OR immigrant* OR emigrant* OR foreigner	"travel"[MeSH Terms] OR "travel"[All Fields] OR "traveling"[All Fields] OR "travelling"[All Fields] OR "travels"[All Fields] OR "traveled"[All Fields] OR "traveler"[All Fields] OR "traveler s"[All Fields] OR "travelers"[All Fields] OR "travelled"[All Fields] OR "traveller"[All Fields] OR "traveller s"[All Fields] OR "travellers"[All Fields] OR ("travel"[MeSH Terms] OR "travel"[All Fields] OR "traveling"[All Fields] OR "travelling"[All Fields] OR "travels"[All Fields] OR "traveled"[All Fields] OR "traveler"[All Fields] OR "traveler s"[All Fields] OR "travelers"[All Fields] OR "travelled"[All Fields] OR "traveller"[All Fields] OR "traveller s"[All Fields] OR "travellers"[All Fields]) OR ("import"[All Fields] OR "importation"[All Fields] OR	193,400

		"importations"[All Fields] OR "imported"[All Fields] OR "importer"[All Fields] OR "importers"[All Fields] OR "importing"[All Fields] OR "imports"[All Fields]) OR "immigrant*"[All Fields] OR "emigrant*"[All Fields] OR ("emigrants and immigrants"[MeSH Terms] OR ("emigrants"[All Fields] AND "immigrants"[All Fields]) OR "emigrants and immigrants"[All Fields] OR "foreigner"[All Fields] OR "foreigners"[All Fields])	
2	(malaria[MeSH Terms]) OR (Plasmodium[MeSH Terms])	"malaria"[MeSH Terms] OR "plasmodium"[MeSH Terms]	89,028
1	((chemoprophylaxis[MeSH Terms]) OR (antimalarials[MeSH Terms])) OR (prophylaxis[MeSH Terms])	"chemoprevention"[MeSH Terms] OR "antimalarials"[MeSH Terms]	49,870

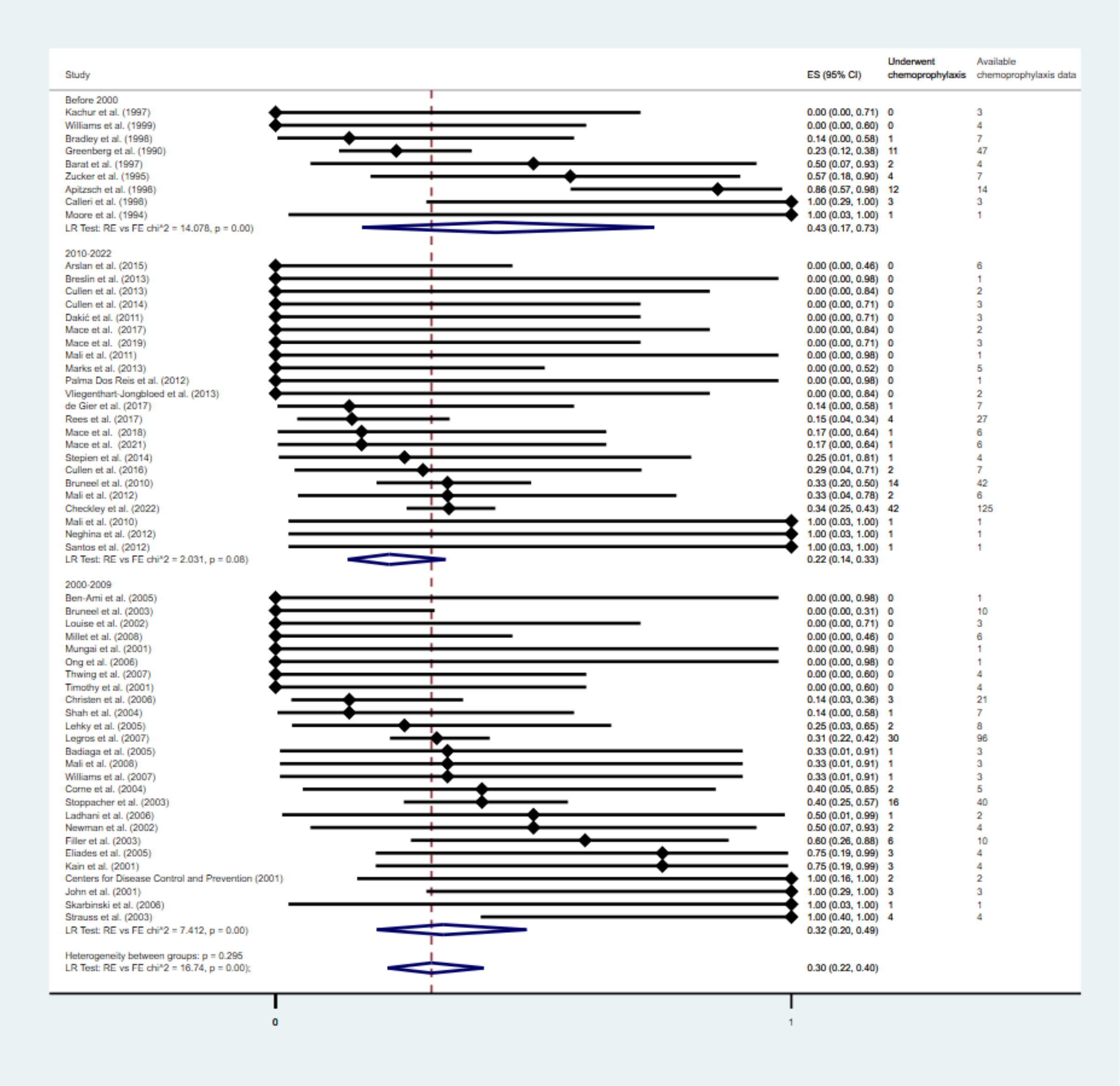

Fig. S1. Subgroup analysis by year of publication showing the pooled proportion of malaria deaths who took any chemoprophylaxis. The subgroups were articles published before 2000, between 2000 and 2009, and between 2010-2022.

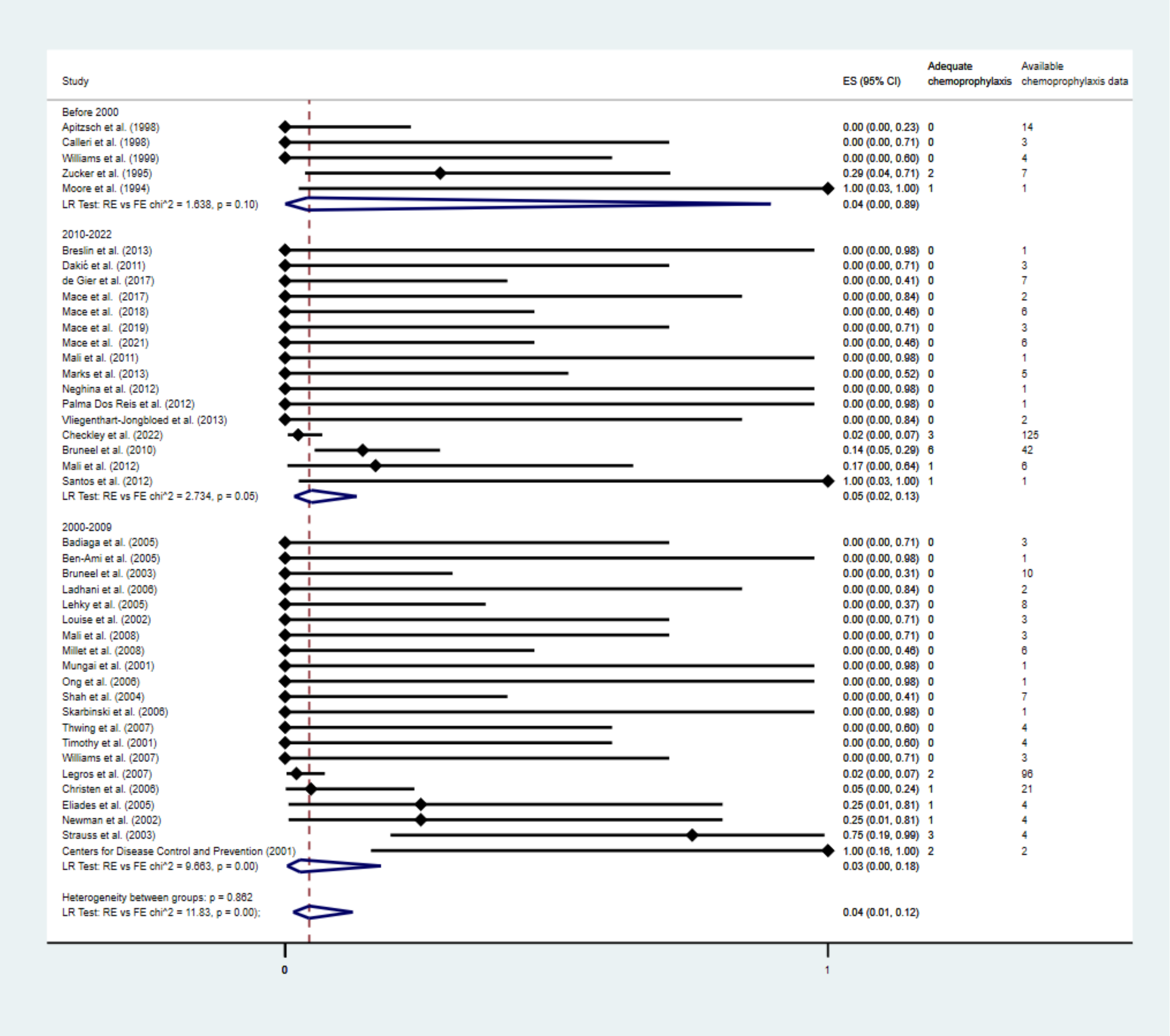

Fig. S2. Subgroup analysis by years of publication showing the pooled proportion of malaria death cases who took adequate chemoprophylaxis. The subgroups were articles published before 2000, between 2000 and 2009, and between 2010-2022.

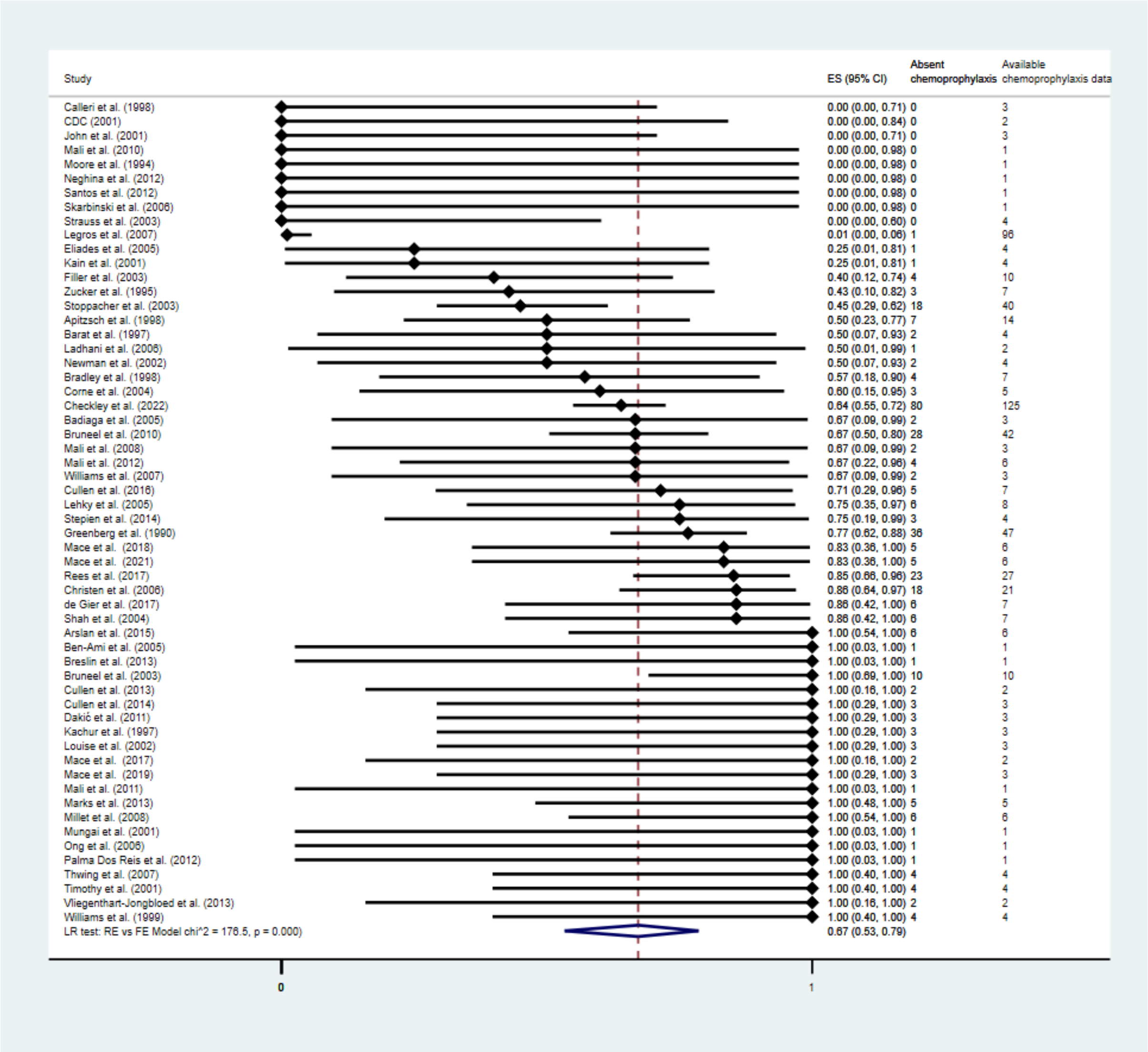

Fig. S3. A meta-analysis demonstrated the pooled proportion of malaria deaths who did not take any chemoprophylaxis.

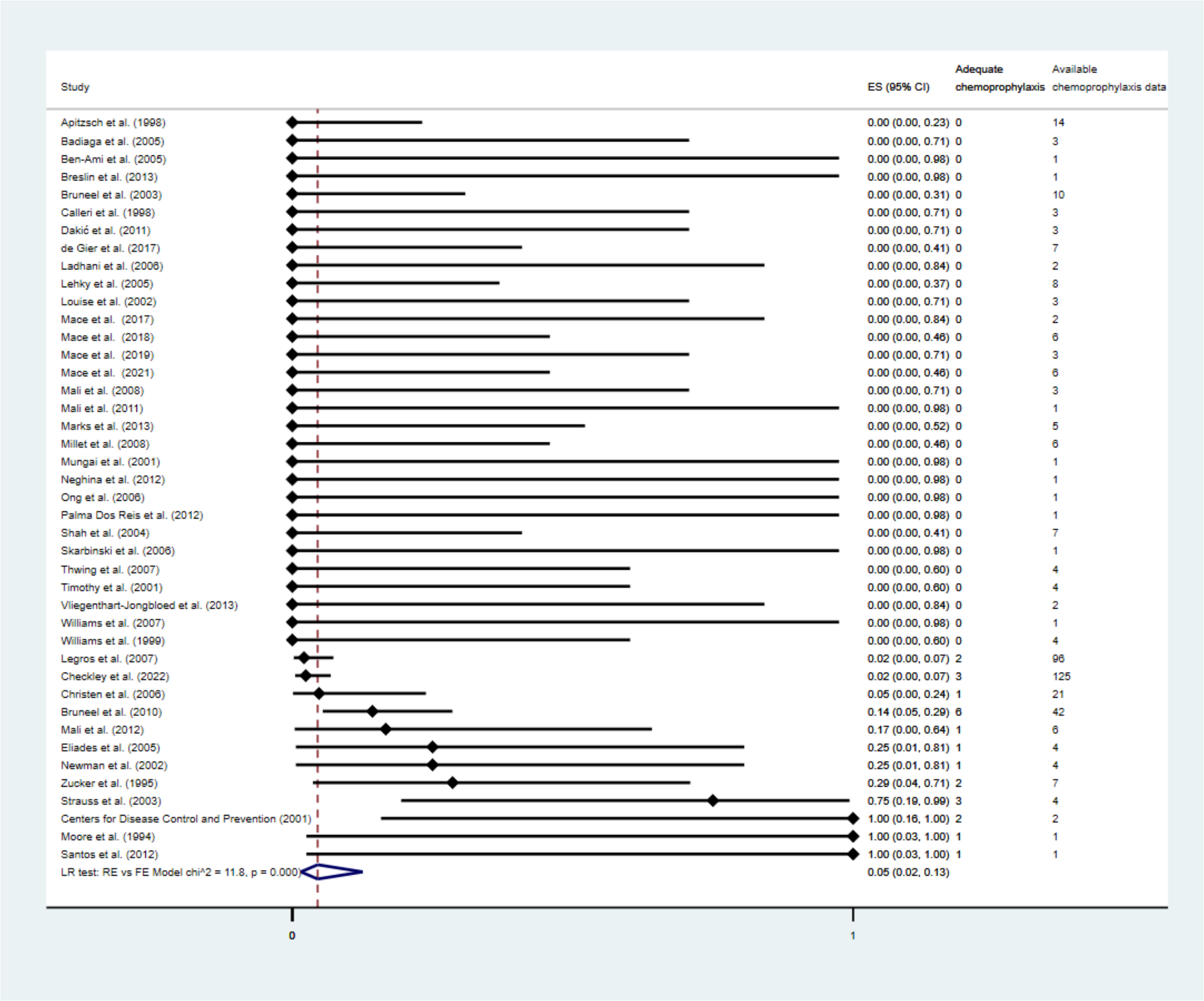

Fig. S4. A meta-analysis showed the pooled proportion of malaria deaths who did not take any chemoprophylaxis.

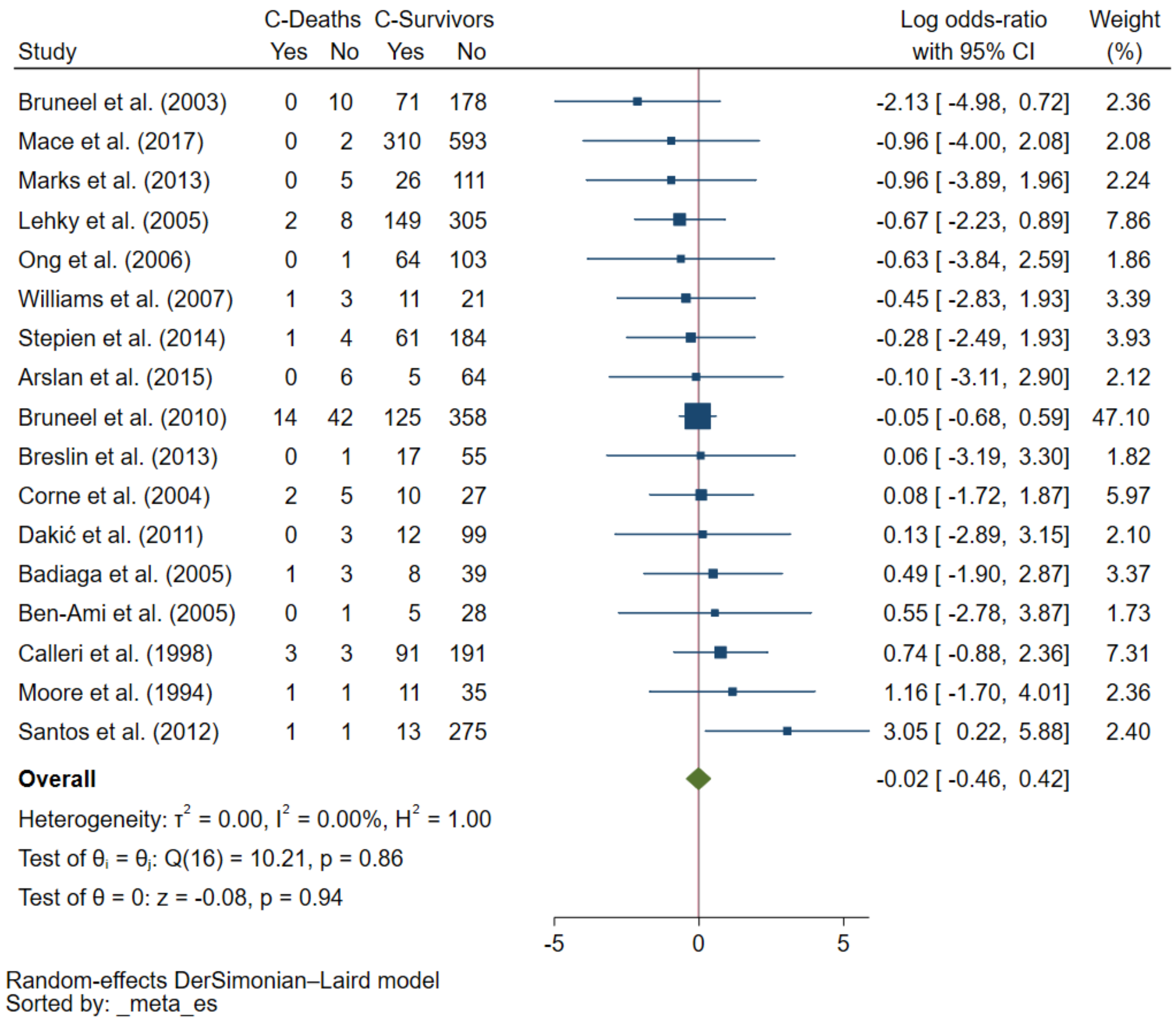

Fig. S5. The meta-analysis results showed that a comparable log-OR of underwent chemoprophylaxis between malaria deaths and survivors. C-Deaths, underwent chemoprophylaxis and deaths; C-Survivors, underwent chemoprophylaxis and survived.

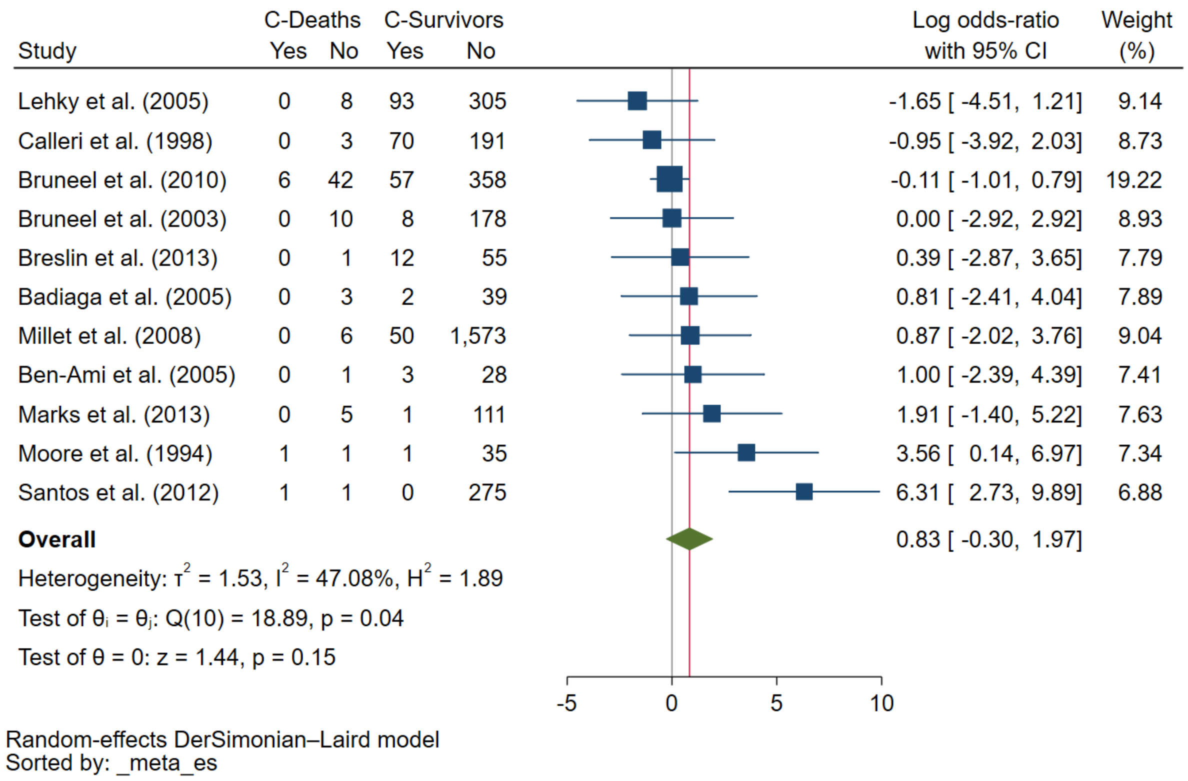

Fig. S6. A meta-analysis demonstrated the pooled log-OR of adequate chemoprophylaxis in malaria deaths and survivors. C-Deaths, underwent chemoprophylaxis and deaths; C-Survivors, underwent chemoprophylaxis and survived.

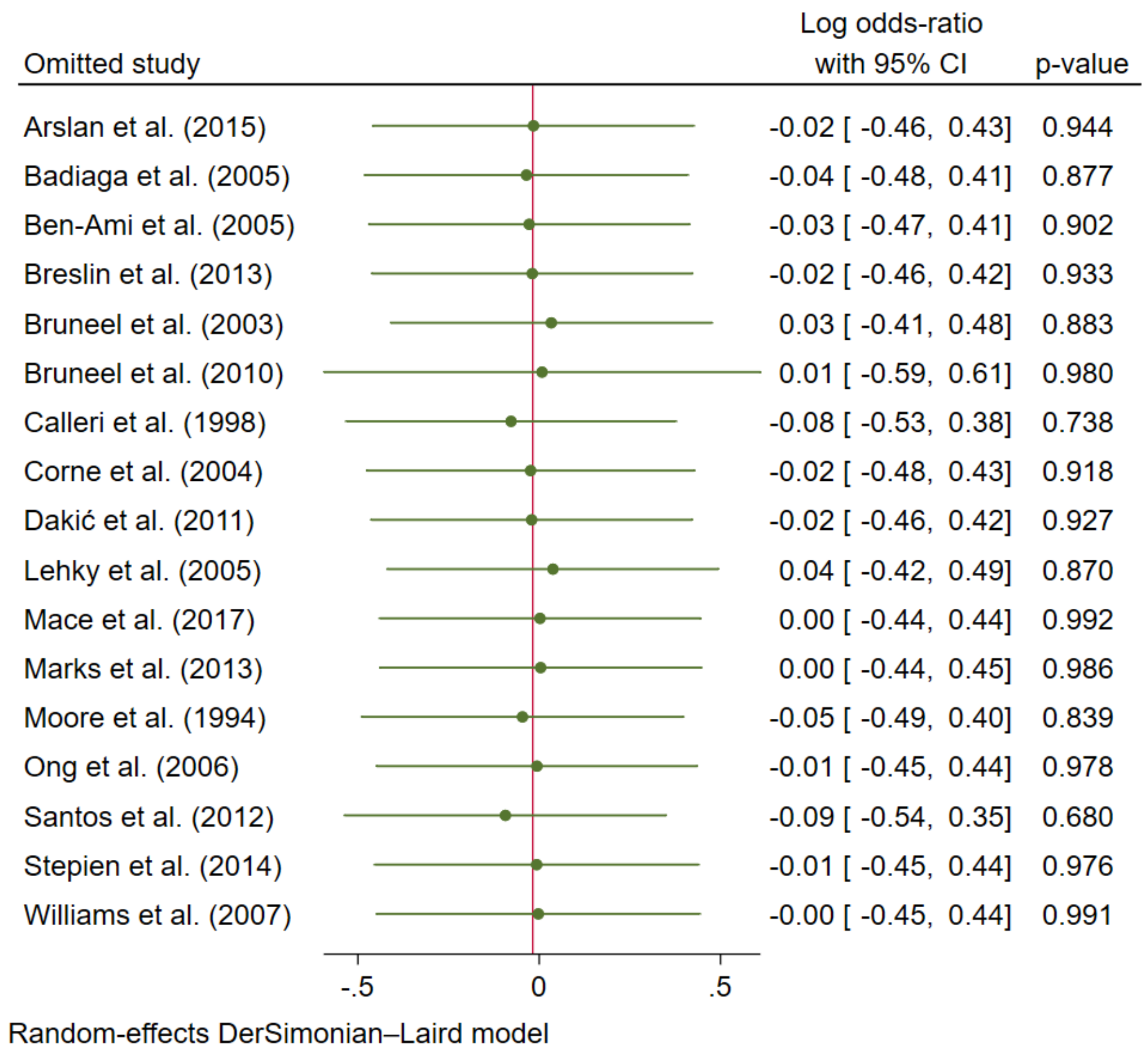

Fig. S7. The leave-one-out method showed that after each study was excluded and re-run the meta-analysis of the pooled log-OR, there was comparable log-OR of underwent chemoprophylaxis between malaria deaths and survivors.

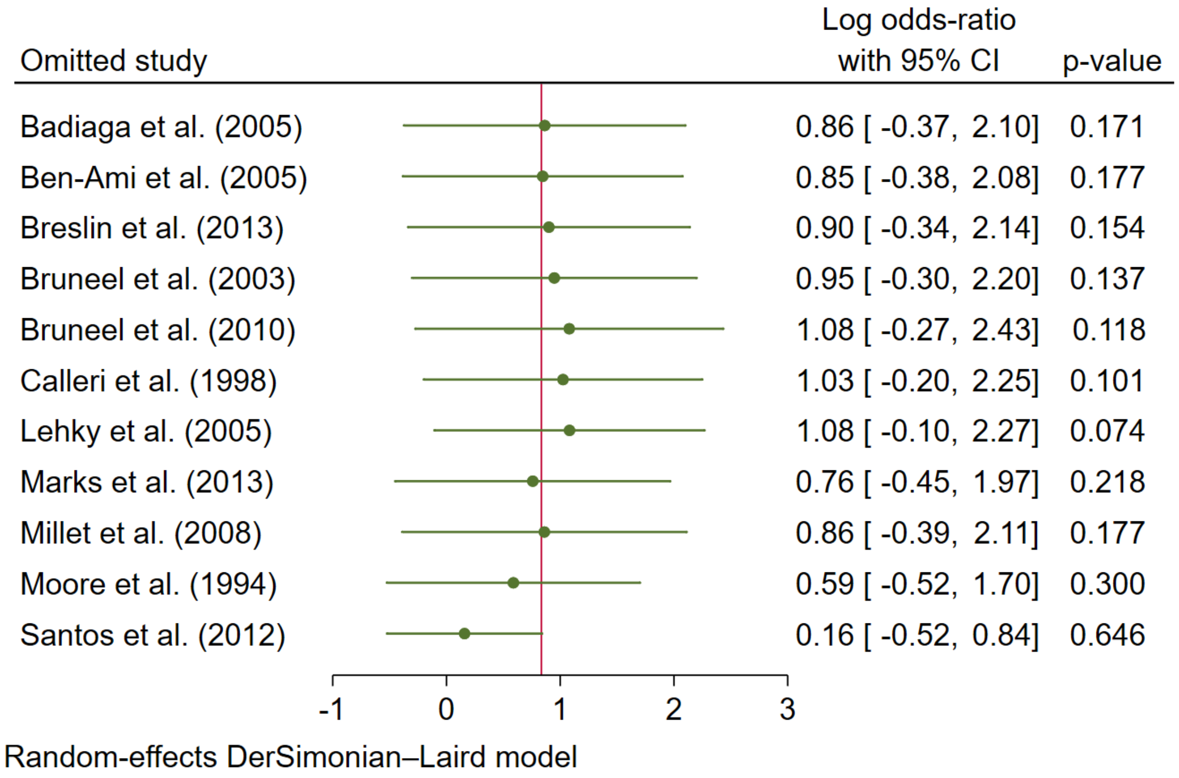

Fig. S8. The leave-one-out method showed that after each study was excluded and re-run the meta-analysis of the pooled log-OR, there was comparable log-OR of adequate chemoprophylaxis between malaria deaths and survivors.

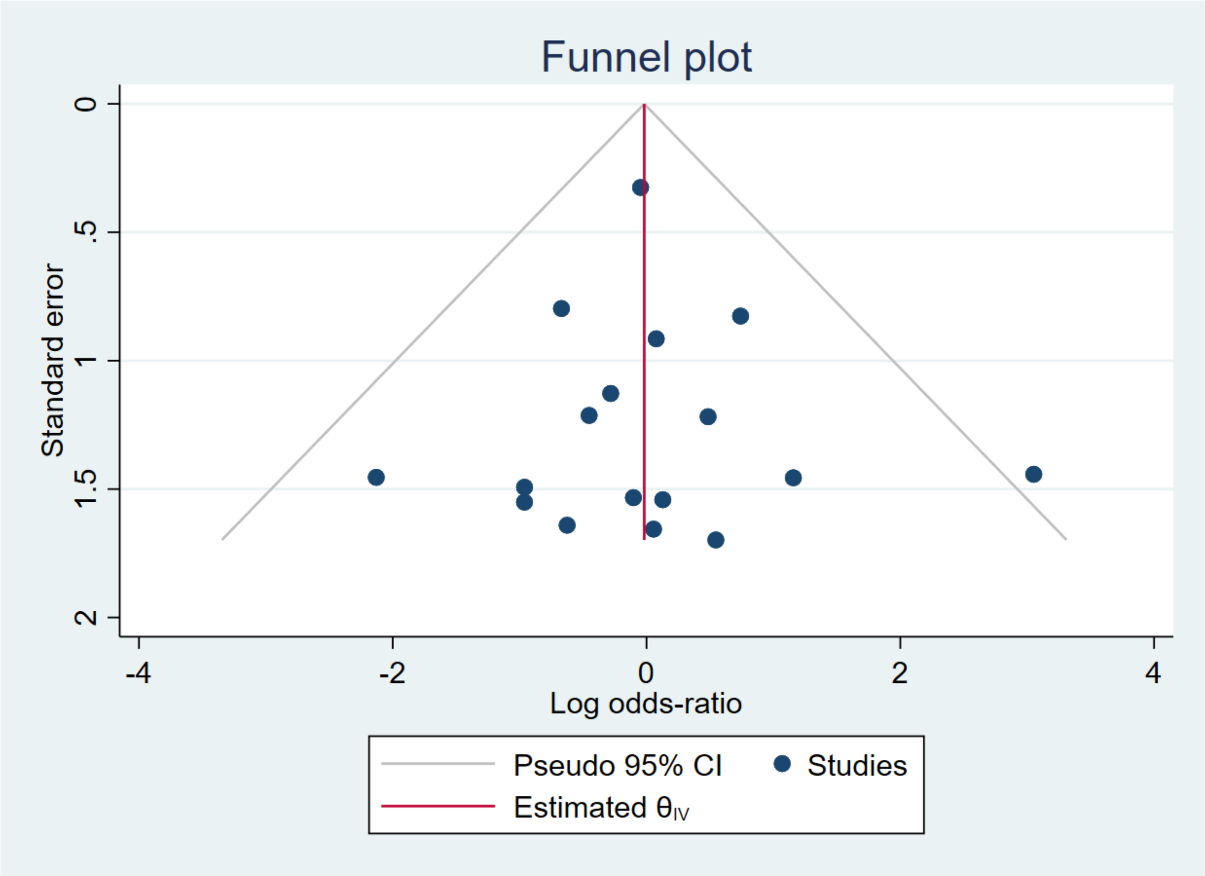

Fig. S9. The funnel plot of the meta-analysis of odds of underwent chemoprophylaxis in deaths and survivors**.**

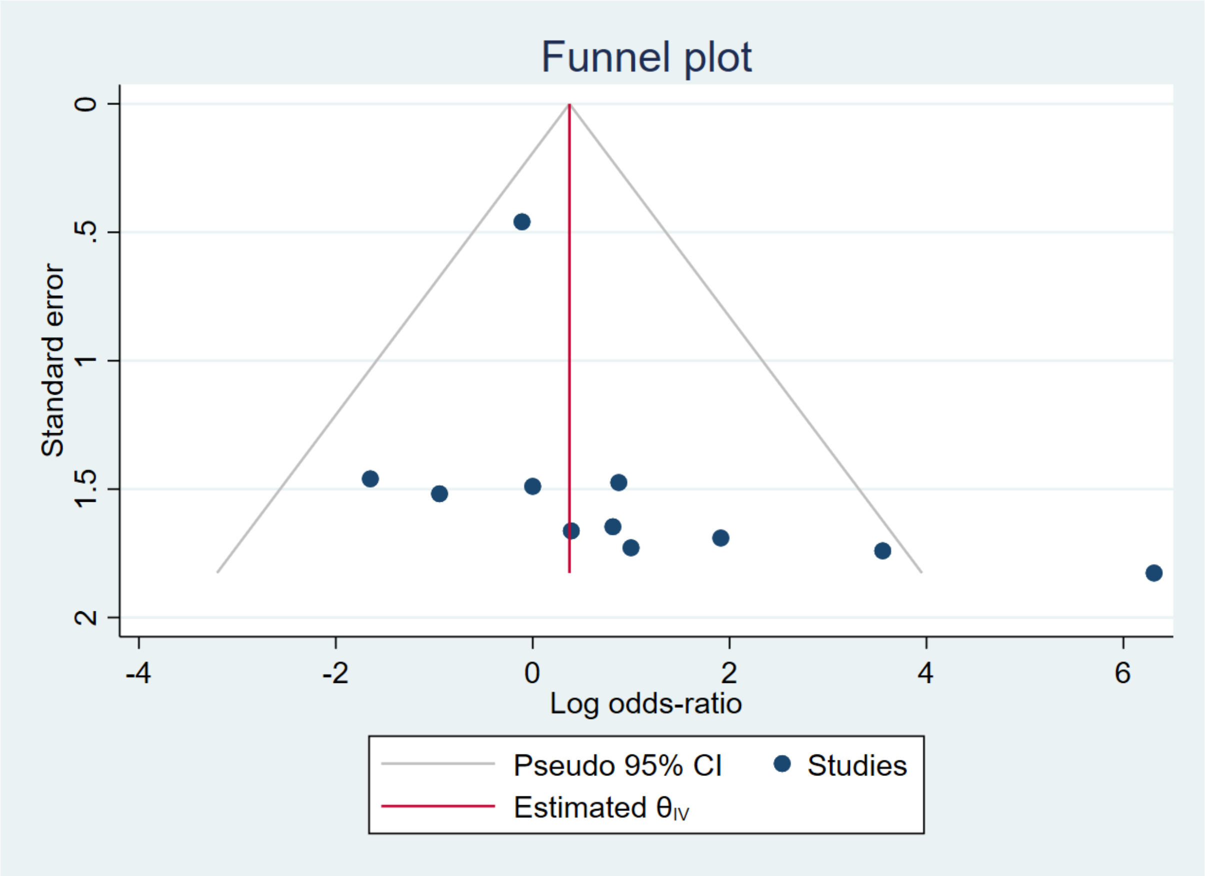

Fig. S10. The funnel plot of the meta-analysis of odds of adequate chemoprophylaxis in deaths and survivors**.**
